# 

**Published:** 1886-11-27

**Authors:** 


PRICE ONE PENNY.

No. 9, Vol. I.
November 27th, 1886.

Per Annum, 6s. 6d., post free.
Monthly Parts, 6d.; post free, 7½d.
Ditto per Ann., 7s. 6d., post free.

